# The Practice of Electroconvulsive Therapy (ECT) in the West of Ireland

**DOI:** 10.1192/bjo.2026.11239

**Published:** 2026-06-30

**Authors:** Titilayo Fadahunsi, Conal Devlin

**Affiliations:** Mayo Mental Health Service, HSE West, Castlebar, Ireland

## Abstract

**Aims:**

This study is aimed at examining the divergent views of mental health staff regarding the use of Electroconvulsive Therapy (ECT) within the Mayo Mental Health Service in the West of Ireland.

**Methods:**

An anonymous online survey was distributed through email to mental health staff in Mayo Mental Health Service across multiple disciplines, including psychology, nursing, medicine, social work, occupational therapy, pharmacy, and CBT/DBT practitioners. Participation was undertaken voluntarily, and no identifying information was collected. The survey remained open for four weeks.

**Results:**

Responses were obtained from 53 staff members, representing nurses (30%), non-consultant hospital doctors (28%), consultants (23%), pharmacists (6%), CBT/DBT practitioners (4%), a psychologist and social worker (2%), and other disciplines (6%). Experience in mental health services varied: 43% had over 15 years, 15% had 10–15 years, 15% had 5–10 years, and 26% had less than 5 years. Most respondents (89%) reported a clear understanding of ECT. In the same vein, 89% would consider ECT for themselves if severely ill, and 79% would support it for a family member if recommended by a specialist. Talking about consent, 72% believed ECT should never be administered without patient agreement. Staff generally viewed ECT as safe (91%) and more effective than other treatments for severe mental illness (92%), though 9% questioned its safety and 8% its efficacy. Opinions on side effects were mixed, with 66% reporting no severe effects and 34% acknowledging potential significant adverse effects.

**Conclusion:**

The results amount to the fact that mental health staff in the West of Ireland generally regard ECT as a safe and effective treatment for severe mental illness. While most staff reported confidence in their knowledge, perceptions of side effects were more variable, underscoring the need for ongoing professional education. The findings also suggest that increasing public awareness of ECT could reduce stigma and misinformation to its barest minimum. Overall, enhancing education for both healthcare professionals and wider community may support informed decision-making and enhance acceptance of this important treatment modality.

